# Reassortment process after co-infection of pigs with avian H1N1 and swine H3N2 influenza viruses

**DOI:** 10.1186/s12917-017-1137-x

**Published:** 2017-07-08

**Authors:** Kinga Urbaniak, Iwona Markowska-Daniel, Andrzej Kowalczyk, Krzysztof Kwit, Małgorzata Pomorska-Mól, Barbara Frącek, Zygmunt Pejsak

**Affiliations:** 1 Department of Swine Diseases, National Veterinary Research Institute, 57 Partyzantów Avenue, 24-100 Puławy, Poland; 2 Present Address: Laboratory of Veterinary Epidemiology and Economics, Faculty of Veterinary Medicine, Warsaw University of Life Sciences, 159c Nowoursynowska Street, 02-776 Warsaw, Poland; 3 Present Address: Wrocław Research Centre EIT+, 147 Stabłowicka Street, 54-066 Wrocław, Poland

**Keywords:** Avian influenza virus, Pig, Reassortment, Swine influenza virus

## Abstract

**Background:**

The influenza A virus is highly variable, which, to some degree, is caused by the reassortment of viral genetic material. This process plays a major role in the generation of novel influenza virus strains that can emerge in a new host population. Due to the susceptibility of pigs to infections with avian, swine and human influenza viruses, they are considered intermediate hosts for the adaptation of the avian influenza virus to humans. In order to test the reassortment process in pigs, they were co-infected with H3N2 A/swine/Gent/172/2008 (Gent/08) and H1N1 A/duck/Italy/1447/2005 (Italy/05) and co-housed with a group of naïve piglets.

**Results:**

The Gent/08 strains dominated over Italy/05, but reassortment occurred. The reassortant strains of the H1N1 subtype (12.5%) with one gene (NP or M) of swine-origin were identified in the nasal discharge of the contact-exposed piglets.

**Conclusions:**

These results demonstrate that despite their low efficiency, genotypically and phenotypically different influenza A viruses can undergo genetic exchange during co-infection of pigs.

**Electronic supplementary material:**

The online version of this article (doi:10.1186/s12917-017-1137-x) contains supplementary material, which is available to authorized users.

## Background

The genetic material of the influenza A virus (IAV) is made up of eight single-stranded, negative-sense RNA segments. Such a genomic arrangement allows the exchange of RNA segments between two or more IAVs that co-infect a single target cell. This process is known as reassortment. It is one of the mechanisms by which new IAVs emerge and spread in immunologically susceptible human as well as swine populations [1, 2].

Pigs have been thought to serve as intermediate hosts in the adaptation of the avian influenza virus (AIV) to humans, and as “mixing vessels” facilitating the formation of novel genetic IAV variants with potentially pandemic properties. This assumption is based on the confirmed ability of pigs to be infected with the swine influenza virus (SIV), AIV and human influenza virus (HuIV) in natural conditions [3, 4]. Furthermore, experimental studies, which confirmed pig susceptibility to infections with the majority of the AIV subtypes emphasise the importance of pigs in the IAV ecology [5–7].

The mechanism of the exchange of genetic material between IAV strains has not been fully elucidated. It is still unclear where reassortment occurs. It may take place in the nucleus during replication, during the export of the RNA from the nucleus to the cell membrane, in the cell cytoplasm after the release of progeny RNA segments, or during progeny virion assembly [8].

Recent studies on the mechanism of IAV genome packaging suggest that the interaction of specific sequences between different RNA segments guides their selective incorporation into the virion [9–16]. Therefore, the reassortment process may be controlled by certain differences in the signals that initiate packing, required for the RNA-RNA interaction of various RNA segments. This was confirmed in the studies of Chou et al. [17] and Inagaki et al. [18], who showed that eight different RNA segments are always packed into one virion [17] and that segments of the same genes compete with each other in the process of virion assembly [18]. Furthermore, the individual segments are packaged in a hierarchical manner. The segments encoding PB2, PA, NP and M protein are more important than the remaining segments. Moreover, RNA segments are not transported from the nucleus individually or in packages of eight [19].

The aim of the study was to evaluate the occurrence of reassortment in piglets co-infected with two influenza viruses of different subtypes and origins. We co-infected the piglets with A/swine/Gent/172/2008 (H3N2) (Gent/08) and A/duck/Italy/1447/2005 (H1N1) (Italy/05). Additionally, healthy piglets were co-housed with the infected piglets.

## Methods

### Study design

For experimental purposes, twelve 6- to 8-week-old piglets were purchased from a high health status herd. They were randomly divided into two groups. Matrix gene real time reverse transcriptase PCR (RT-qPCR) and the haemaglutynation inhibition assay with four SIV subtypes: avian-like H1N1 (A/swine/Belgium/1/98), human-like H1N2 (A/swine/England/96), human-like H3N2 (A/swine/Flanders/1/98) and pdm-like H1N1 (A/swine/Poland/031951/2012), performed according to the standard procedure [20], were carried out prior to commencing the study. All tested piglets were molecularly and serologically negative for SIV. During the study, the piglets were housed in isolated units.

Two viral strains, the Gent/08 and the Italy/05, were propagated in 10-day-old embryonated chicken eggs. The third and fourth passage of the virus was used in a 1:1 mixture. A 10^9^ TCID_50_ IAV mixture was administered intranasally (i.n.) at a volume of 2 ml per nostril to a group of six piglets (forced-infected, FI). The remaining six naïve piglets formed the contact-exposed (CE) group and were introduced to the FI animals in the second day post infection (dpi).

### Clinical measurements and sampling

During the study (10 dpi/day post contact (dpc)), the clinical signs of swine influenza (SI) were assessed daily. This included collecting swab samples and monitoring body temperature. At 4 dpi/dpc three randomly selected piglets from both groups were euthanised and necropsied. Blood samples were collected from all of the piglets before the initiation of the study and on the 14th, 21st, and 28th dpi/dpc for a serological investigation.

### Swabs and tissue samples

Nasal swabs and tissue samples (of the respiratory and olfactory nasal mucosa, the trachea, the right and left apical, cardiac, diaphragmatic and accessory lobe of the lungs) were collected and prepared for extraction of viral RNA using the QIAmp Viral RNA Mini kit (Qiagen, Germany). The obtained samples were assessed using the RT-qPCR method, as described below.

### RT-qPCR

The sequences of primers and the probe were acquired from Hoffmann et al. [21]. A single RT-qPCR was performed using 2 μl of the template RNA, 1× QuantiTect Probe RT-PCR Master Mix, 0.2 μl QuantiTect RT Mix (Qiagen, Germany), 0.8 μM of the forward primer, 0.6 μM of each reverse primer and 0.1 μM of the probe with the addition of water free from DNase and RNase to bring the total volume to 20 μl.

All the RT-qPCR assays were performed on a Stratagene MX3005P QPCR thermocycler (Agilent Technologies, USA) using the following temperature profile: 30 min at 50 °C, 15 min at 95 °C, 40 cycles of 10 s at 94 °C and 20 s at 60 °C. The fluorescence values (FAM) were collected during the annealing step. Reactions with a cycle threshold (Ct) value of <30 were scored positive, values between 30 and 35 were scored as weak positive and values >35 were scored negative.

### Plaque purification assay

All the positive and weakly positive nasal swabs as well as the tissue samples were used in the plaque purification assay to obtain single IAV isolates.

Six well plates with 100% confluent MDCK culture were washed once with PBS. The inoculum (500 μl of the sample dilution) was applied to the wells of the plates. The infected cells were incubated at 37 °C for 1 h in 4% CO_2_. Then, the inoculum was removed and 3 ml overlays of Eagle’s MEM with an addition of 0.9% agarose and 1 μg/ml trypsin were added. The plates were placed inversely in a 37 °C, humidified incubator containing 5% CO_2_. The plaques were visualised 2 days post infection. The agar was punctured where the cells were damaged by the replicating virus. The agar fragments were suspended in 500 μl of Eagle’s MEM and then 5-fold diluted. Subsequently, the diluted samples were passaged once on MDCK cells. A cytopathic effect and RT-qPCR confirmed the presence of IAV. The obtained virus isolates were used to extract viral RNA or were stored at −80 °C for further analysis.

### Molecular characterisation

Numerous conventional reverse transcriptase PCRs (RT-PCRs) (Additional file 1: Table S1) were carried out to genetically differentiate Gent/08 and Italy/05. RNA samples from the plaque-purified IAVs were used. The primers were selected based on the nucleotide sequence of parental IAV genomes using version 0.4.0 of the Primer3 Input (http://bioinfo.ut.ee/primer3-0.4.0/) and the PrimerSelect package LaserGene DNASTAR application. The primers for 6 RT-PCRs were used in order to amplify the products of the internal IAV genes (PB2, PB1, PA, NP, M, NS). The products were then used in the analysis of restriction fragment length polymorphism (RFLP). The remaining 4 pairs of primers were specific for the Gent/08 or Italy/05 HA and NA (Additional file 1: Table S1). RT-PCRs were performed separately using the One Step RT-PCR Kit (Qiagen, Germany).

All the primer sets were used in a single RT-PCR as follows: 2.5 μl of the template RNA, 1× OneStep RT-PCR Buffer, 1 μl of the OneStep RT-PCR Enzyme Mix containing reverse transcriptase and DNA polymerase (Qiagen, Germany), 0.5 mM of each of the dNTPs, 10 U of the RNaze inhibitor (Promega, USA) and the primers (0.5 μM of each primer) were combined and water free from DNase and RNase was added to bring the total volume to 25 μl.

The following temperature profiles were used for all the RT-PCR runs: 30 min at 50 °C, 15 min at 95 °C, 45 cycles of 45 s at 94 °C, 45 s at 58 °C, and depending on the length of the amplified product, from 1.5 to 5 min at 72 °C. The last cycle was followed by a 7-min extension at 72 °C. After the amplification, 10 μl of the product was subjected to electrophoresis for 40 min at 300 mA in a 2% agarose gel stained with ethidium bromide. The amplification products were visualised and photographed under UV illumination using the EC3 Chemi HR 410 Imaging System (Ultra-Violet Products Ltd., UK).

Restriction enzymes (BamHI, NheI and EcoRI) were selected for the RFLP analysis using version 3 of RestrictionMapper (http://www.restrictionmapper.org/).

The cleavage reaction was performed in a final volume of 16 μl containing 5 μl of the amplified product, 1 μl of the adequate restriction enzyme (Additional file 1: Table S2) and 1 μl of the 10× Buffer (Thermo Scientific, USA), completed with the water free from DNase and RNase.

The enzymes were activated for 2 h at 37 °C, then inactivated for 20 min at 65 °C (*Nhe*I, *Eco*RI) or 80 °C (*Bam*HI). All the RT-PCRs and cleavage reactions were performed using the T3 Thermocycler (Biometra, Germany). After cleavage, the product was subjected to electrophoresis as described above.

### Sequencing analysis

The amplified RT-PCR products were sequenced in Genomed S.A., a DNA analysis service (Poland). The nucleotide sequences were initially compared using the ClustalW alignment algorithm method.

## Results

### Clinical signs

Apart from an increase in the rectal temperature, there were no typical clinical signs for acute SI either in the FI or the CE group of piglets.

In all the FI animals, the highest rectal temperature (40.0 °C - 40.8 °C) in individual piglets lasted from 2 to 5 dpi, with modal value at 3 dpi. A rectal temperature ≥ 40 °C was observed in three of the six FI piglets on days 2 and 3. In the CE piglets, a rectal temperature ≥ 40 °C was detected only in two piglets (peak from 3 to 4 dpc) and did not exceed 40.2 °C (data not shown).

### Virus detection

In total, 56 nasal swabs and tissue samples were collected with a Ct value ≤35. Seventeen of the nasal swab samples were positive and 10 were weakly positive (Table 1). Nineteen tissue samples were positive and 10 samples were weakly positive (Table 2).

**Table 1:** RT-qPCR results for nasal swabs of the FI and the CE piglets

Dpi/dpc	Forced-infected piglet ID						Contact-exposed piglet ID					
	1	2	3	4	5	6	A	B	C	D	E	F
0	−	−	−	−	−	−	−	−	−	−	−	−
1	−	−	++	−	−	++	+	−	−	−	−	−
2	+	−	++	+	+	++	−	−	−	−	−	−
3	+	−	++	−	++	++	−	+	++	−	−	++
4	−	−	++	-^a^	+^a^	++^a^	−	++	++	-^a^	-^a^	++^a^
5	−	−	+	×	×	×	−	++	++	×	×	×
6	−	+	+	×	×	×	−	−	++	×	×	×
7	−	−	−	×	×	×	−	−	−	×	×	×
8	−	−	−	×	×	×	−	−	−	×	×	×
9	−	−	−	×	×	×	−	−	−	×	×	×
10	−	−	−	×	×	×	−	−	−	×	×	×

Results are shown as ++ for positive sample (Ct < 30), + for weak positive sample (Ct 30–35) and - for negative sample (Ct > 35)

^a^ euthanasia

**Table 2:** RT-qPCR results for tissue samples of the FI and the CE piglets

Tissue sample	Forced-infected piglet ID			Contact-exposed piglet ID		
	4	5	6	D	E	F
Respiratory nasal mucosa	−	++	++	−	−	++
Olfactory nasal mucosa	−	++	++	−	−	++
Trachea	+	+	++	++	+	++
Right apical and cardiac lobe	++	++	−	++	−	++
Right diaphragmatic lobe	−	+	−	++	−	++
Left apical and cardiac lobe	−	+	−	−	−	++
Left diaphragmatic lobe	−	+	+	++	+	++
Accessory lobe	−	+	−	−	+	++

Results are shown as ++ for positive sample (Ct < 30), + for weak positive sample (Ct 30–35) and - for negative sample (Ct > 35)

**Fig. 1:**
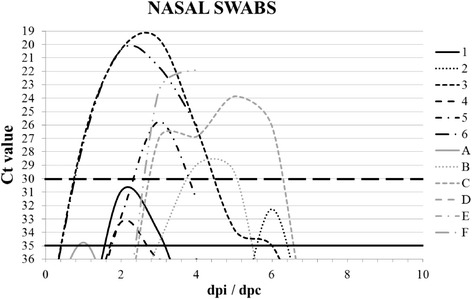
Viral shedding by the FI and the CE piglets. 1–6 – FI piglets; A-F – CE piglets

Viral shedding was confirmed for all the FI animals, but only three piglets (Nos. 3, 5 and 6) shed the virus extensively (Ct < 30). In addition, Ct values of the nasal swabs of piglets no. 3 and 6 were <30 and ranged from 1 to 4 dpi. In the CE group, viral RNA was detected in swab samples from four of the six piglets (piglets A, B, C and F). However, three of them shed the virus extensively (Ct < 30), for 2 or 4 days (from 3 to 6 dpc), depending on the animal (Fig. 1).

Seven positive and 7 weakly positive tissue samples were collected from the euthanised FI piglets. In each case, samples from trachea gave a positive or a weakly positive result. In the remaining tissues, positive or weakly positive results were obtained in 1 or 2 specimens. The samples from the upper respiratory tract as well as the right apical and cardiac lung lobe (pooled samples) showed a Ct value <30. All the samples from pig no. 5 gave a positive result (Table 2).

In the case of the CE animals, 12 positive and 3 weakly positive tissue samples were obtained. All the tested trachea and left diaphragmatic lobe samples from the CE piglets were positive or weakly positive. The lung samples representing the right apical and cardiac lobe (pooled samples), the right diaphragmatic lobe and the accessory lobe gave a positive reaction in two animals. The tissue samples of the respiratory tract from piglet F were positive (Table 2).

### Plaque purification assay

Plaque-purified IAV isolates were obtained from 26 tissue samples. The arithmetic mean of the number of the isolates obtained from the samples with Ct values from 30 to 35 was 7.00 ± 5.15, and was equal to 8.11 ± 4.01 for samples with a Ct < 30. The total number of isolates obtained from the tissue homogenates was 202. One hundred thirty-one of those isolates were acquired from tissues of the CE animals and 71 came from the tissues of the FI piglets.

Twenty-six plaque-purified IAV isolates (24 from the CE piglets, 2 from the FI piglets) were obtained from 7 nasal swab samples (6 from the CE piglets, 1 from the FI animal). The arithmetic mean of the number of isolates obtained from the nasal swabs was 2.67 ± 1.37 with exclusion the sample from piglet F from the CE group, as 10 isolates were collected from this animal.

### Molecular characterisation

All 228 obtained isolates were used for the genetic characterisation. Amplicons specific for the HA (463 bp) and the NA (241 bp) Gent/08 genes were obtained for 220 isolates (96.49%) (H3N2 isolates), including 202 from the tissue samples and 18 from the nasal swabs. Results specific for the Italy/05 genes (HA – 314 bp, NA – 327 bp) were obtained for the remaining 8 isolates (H1N1 isolates) from the nasal swabs (Tables 3 and 4).

**Table 3:** Genetic makeup of plaque-purified IAVs based on the RT-PCR and the RFLP analyses

Isolate	Gene origin	Gene							
		PB2	PB1	PA	HA	NP	NA	M	NS
H1N1	Italy/05	8	8	8	8	6	8	7	8
	Gent/08	0	0	0	0	**2**	0	**1**	0
H3N2	Italy/05	0	0	**1**	0	**1**	0	0	0
	Gent/08	220	220	219	220	219	220	220	220

Results are shown as the number of genes. Samples for sequencing shown in bold

**Table 4:** Plaque-purified IAVs from tissue and nasal swabs samples from the FI and the CE piglets

Virus	Forced-infected		Contact-exposed		In total
	Tissue samples	Nasal swabs	Tissue samples	Nasal swabs	
H3N2	100 (71/71)	100 (2/2)	100 (131/131)	66.67 (16/24)	96.49 (220/228)
H1N1	-	-	-	20.83 (5/24)	2.19 (5/228)
rH1N1^a^	-	-	-	12.50 (3/24)	1.32 (3/228)

Results are shown as percentages, with the number of viruses/total number of viruses shown in parentheses

^a^Reassortant H1N1

The *Bam*HI, *Nhe*I and *Eco*RI restriction enzymes were used to differentiate six internal genes. According to the RFLP analysis, the PB2, PB1, M and NS genes of all H3N2 isolates were swine-origin. Two H3N2 isolates had one gene (PA or NP) positive for Italy/05, while the remaining H3N2 isolates had swine-origin PA and NP genes. In the case of the H1N1 isolates, all PB2, PB1, PA and NS genes were of avian-origin. Three H1N1 isolates had one gene (NP or M) with a Gent/08 specific cleavage pattern (Table 3).

### Sequencing

A gene fragment (RT-PCR amplification product) sequence analysis was conducted for the RNA samples selected according to the RFLP results. In total, 5 samples (marked in bold in Table 3) were analysed. A mutation at the *Bam*HI cleavage site of the PA and the NP gene was identified in two H3N2 isolates positive in the RFLP for Italy/05 genes. The sequence analyses of 3 genes (M or NP) of swine-origin H1N1 isolates confirmed the RFLP results.

### Analysis of molecular results

All 202 isolates obtained from the tissue samples were infected by the H3N2 subtype. Of the 26 isolates collected from the nasal swabs, 18 were Gent/08, 5 were Italy/05, and the remaining 3 were reassortants (H1N1 subtype with one gene of swine-origin, rH1N1). All the H1N1 and 16 Gent/08 strains were obtained from swab samples from the CE animals. Two Gent/08 isolates were from the nasal swab collected from the FI piglet no. 6 (Table 4). Each reassortant was obtained from the samples collected from different CE animals (Table 5).

**Table 5:** Molecular characteristics of the obtained rH1N1

Piglet ID / Sampling day	Subtype	Molecular characterisation	Reassortant
C / 5dpc	H1N1	PB2, PB1, PA, HA, NP, NA, **M** ^a^, NS	r7
E / 4dpc	H1N1	PB2, PB1, PA, HA, **NP** ^a^, NA, M, NS	r5
F / 1dpc	H1N1	PB2, PB1, PA, HA, **NP** ^a^, NA, M, NS	r5

^a^ genes of swine-origin

## Discussion

The emergence of new IAV in humans, pigs and other host species is mostly associated with the reassortment process of two or more IAV strains [22–24]. This study intended to clarify the role of pigs in this process. Our data confirmed that pigs can be experimentally co-infected with SIV and AIV and that an exchange of RNA segments may occur.

The vast majority of the isolated strains were Gent/08 (96.49%). The Italy/05 strain comprised 2.19% of the isolates, while the rH1N1 strain was confirmed in 1.32% of the isolates. All of the Italy/05 and rH1N1 strains were isolated from the nasal swabs of different CE pig.

The transmission of the Italy/05 virus in pigs confirmed in our study strongly contrasts with previous studies which showed efficient replication of this virus in pigs which was not transmitted further [5]. In our experiment, the Italy/05 could be transmitted. Moreover, our rH1N1 possessed a majority of avian-origin genes excluding the M or NP genes. It was reported that the M and NP genes have an impact on the transmission capability of IAV [25–27]. In the case of the r5 H1N1 isolates, the reassortment process may have occurred independently in two CE piglets or, presumably, in one CE or one FI piglet, which then transmitted the r5 H1N1 to naïve animals.

A study conducted in the 1990s indicated that SIV acts as a helper virus in the process of AIV replication in pigs. In that study, the animals were co-infected with SIV Sw/Hok/2/81 (H1N1) and AIV Dk/Hok/8/80 (H3N8), which showed no ability to replicate in the pig. Apart from SIVs, AIVs and rH3N1 with swine-origin NA, NP and M or NS genes were obtained [7]. The SIV used by the Japanese researchers supported the replication of the AIV, whereas in our study, SIV strongly competed with AIV. Nonetheless, in our study, the infection with Gent/08 and the clinical symptoms such as sneezing and coughing may have facilitated the transmission of Italy/05.

An antigenic shift did not occur in our study and the rH1N1 gained only one RNA segment. This could be connected with competitiveness among the used strains.

All of the rH1N1 were isolated from nasal swabs. This may suggest favourable conditions in the upper respiratory tract for the exchange of IAV RNA segments. A similar conclusion was drawn from the experimental co-infection of ferrets with A/Wyoming/03/03 (H3N2) (low virulence, high transmissivity) and A/Thailand/16/06, (avian-like H5N1) (high virulence, low transmissivity). In that study, a low reassortment (8.9%) efficiency was obtained [28], which is similar to our findings. This may be caused by phenotypic and genotypic differences of the IAVs used for co-infection.

The ability of IAV to infect different hosts largely depends on the optimal gene constellation [29]. Thus, the capacity for replication and transmission in pigs may increase in the case of reassortants that have compatible genes compared with a wholly AIV. However, without an experimental comparison we cannot assume that our rH1N1 isolates are better adapted to pigs than Italy/05.

In previous studies, 63 possible reassortants of the H5N1 subtypes of human seasonal A/Wyoming/3/2003 (H3N2) and avian-like A/Thailand/16/2004 (H5N1) IAVs were divided into 4 phenotypic groups based on the rescue efficiency. Strains with a similar genomic constellation to our rH1N1 were included in group 1 (r7 H5N1), while strains with good replication efficiency and marginally viable reassortants made up group 4 (r5 H5N1). The authors underlined that all of the strains in group 4 had an NP gene of mammalian-origin, but the addition of an NS gene (r5/8) or NS and M genes (r5/7/8) from the H3N2 virus significantly increased the rescue efficiency of the reassortant [30].

Another research group generated 254 reassortants of a low pathogenic AIV H5N1 subtype and a HuIV H3N2 subtype [1]. In that study, the strains with M or NP gene of mammalian-origin were included in group 1 (the M gene) and 4 (the NP gene).

Both studies [1, 30] showed that the acquisition of the M gene of mammalian-origin did not significantly affect the functionality of the AIV, while the presence of the NP gene dramatically decreased the activity of the virus. Those results differ from our findings, because 2 of the 3 obtained rH1N1 strains acquired the NP gene. This difference may result from the use of different strains of AIV. It may also be caused by better compatibility of the Gent/08 NP gene with the remaining Italy/05 RNA segments and the congruity of Gent/08 NP with other proteins, especially viral polymerase.

The acquisition of a single gene by the rH1N1 in this study did not increase the frequency of its isolation compared with Italy/05. On the other hand, the results indicate that the rH1N1 viruses have not lost the capability to replicate. Taking into consideration the results of the previous studies [30], we can assume that acquiring another gene of swine-origin could improve the replication and transmission properties of our rH1N1 and enhance its adaptation in pigs.

The most favourable conditions for the occurrence of reassortment during co-infection in experimental models have not been determined. Therefore, it is not clear what circumstances are needed under natural conditions to promote the emergence of new IAV variants [8].

In order to exclude certain unfavourable reassortment factors, such as segment mismatch, an American research group [8] used for co-infection the H3N2 subtype IAV strains with a different genotype. Even then, a theoretically optimal reassortment efficiency was not achieved. They found that parental strains occurred more frequently than expected, suggesting incomplete mixing of RNA segments of parental viruses in co-infected cells, although this may also be related to the small differences in the input of viral particles into single cells [8]. The authors also found that in vivo reassortment occurred less frequently (59%) than in vitro reassortment (88.4%) [8].

Therefore, the use of two phenotypically and genotypically different strains in our experiment had a significant impact on the low incidence of reassortment, mainly due to RNA segment mismatch and the competitiveness of the Gent/08 and the Italy/05 strains. The low incidence of the reassortment was also affected by the use of an animal model.

In previous in vivo studies [31], pigs were inoculated with two SIV strains: the classical H1N1 (cSIV) and the triple reassortant H3N2. The majority (85.9%) of the obtained isolates were reassortants. Interestingly, there were no cSIV strains among all the isolates. This may indicate that the cSIV strain replicates less efficiently in pigs compared to the H3N2 SIV strain and the identified reassortants. Despite such a high percentage of reassortants, their transmission to the CE pigs was not confirmed [31].

The results of that study suggest that there was Italy/05 and Gent/08 reassortment in the CE piglets in our study. This is due to the fact that despite the use of two SIV strains by the American researchers, their reassortants were not transmissible. However, as mentioned above, the confirmed transmission of both IAVs used in our experiment dose not exclude the possibility of reassortant transmission.

Provided there are optimal conditions for reassortment, segment exchange is influenced by the viral strains. The functional compatibility of the synthesised proteins, the match between the sequences initiating RNA segment packing to progeny virions [12] and/or the efficiency of co-infection impacts in the affinity for various receptors [32].

The results of this study show that despite the very low efficiency of reassortment, it can occur between genotypically and phenotypically different IAV strains in pigs. This underscores the necessity for enhanced viral surveillance strategies that monitor reassortment events, as well as for experimental studies which will elucidate the the reassortment process.

## Conclusions

Despite the low efficiency a reassortment between genotypically and phenotypically different influenza A virus strains can occur in pigs.

Infection with swine influenza A virus can help in the transmission of less adapted avian influenza virus.

## Additional file

Additional file 1: Table S1. Contains the names and sequences of all primers used to amplify fragments of all genes to differentiate gene segments of the Gent/08 and the Italy/05. **Table S2.** Contains the information on which gene of which strain (Italy/05 or Gent/08) is subject to restriction enzyme cleavage. It also provides the information about the size of the RT-PCR products and the fragments after the restriction enzyme digestion. (DOCX 14 kb)

## Abbreviations

AIV: Avian influenza virus
CE: Contact-exposed
cSIV: Classical swine influenza virus, subtype H1N1
Ct: Cycle treshold
dpc: Day post contact
dpi: Day post infection
FI: Forced-infected
Gent/08: A/swine/Gent/172/2008 subtype H3N2
HuIV: Human influenza virus
i.n.: Intranasally
IAV: Influenza A virus
Italy/05: A/duck/Italy/1447/2005 subtype H1N1
r: reassortants
RFLP: Restriction fragment length polymorphism
RT-PCR: Reverse transcriptase PCR
RT-qPCR: Real time reverse transcriptase PCR
SI: Swine influenza
SIV: Swine influenza virus

## References

[1] Li C, Hatta M, Nidom CA, Muramoto Y, Watanabe S, Neumann G, Kawaoka Y (2010). Reassortment between avian H5N1 and human H3N2 influenza viruses creates hybrid viruses with substantial virulence. Proc Natl Acad Sci U S A.

[2] Urbaniak K, Markowska-Daniel I (2014). In vivo reassortment of influenza viruses. Acta Biochim Pol.

[3] Nelson MI, Wentworth DE, Culhane MR, Vincent AL, Viboud C, LaPointe MP, Lin X, Holmes EC, Detmer SE (2014). Introductions and evolution of human-origin seasonal influenza a viruses in multinational swine populations. J Virol.

[4] Nelson MI, Vincent AL (2015). Reverse zoonosis of influenza to swine: new perspectives on the human-animal interface. Trends Microbiol.

[5] De Vleeschauwer A, Van Poucke S, Braeckmans D, Van Doorsselaere J, Van Reeth K (2009). Efficient transmission of swine-adapted but not wholly avian influenza viruses among pigs and from pigs to ferrets. J Infect Dis.

[6] Hinshaw VS, Webster RG, Easterday BC, Bean WJ (1981). Replication of avian influenza a viruses in mammals. Infect Immun.

[7] Kida H, Ito T, Yasuda J, Shimizu Y, Itakura C, Shortridge KF, Kawaoka Y, Webster RG (1994). Potential for transmission of avian influenza viruses to pigs. J Gen Virol.

[8] Marshall N, Priyamvada L, Ende Z, Steel J, Lowen AC. Influenza virus reassortment occurs with high frequency in the absence of segment mismatch. PLoS Pathog. 2013; doi:10.1371/journal.ppat.1003421. 10.1371/journal.ppat.1003421 PMC3681746 23785286

[9] Fournier E, Moules V, Essere B, Paillart JC, Sirbat JD, Isel C, Cavalier A, Rolland JP, Thomas D, Lina B, Marquet R (2012). A supramolecular assembly formed by influenza a virus genomic RNA segments. Nucleic Acids Res.

[10] Fujii Y, Goto H, Watanabe T, Yoshida T, Kawaoka Y (2003). Selective incorporation of influenza virus RNA segments into virions. Proc Natl Acad Sci U S A.

[11] Fujii K, Fujii Y, Noda T, Muramoto Y, Watanabe T, Takada A, Goto H, Horimoto T, Kawaoka Y (2005). Importance of both the coding and the segment-specific noncoding regions of the influenza a virus NS segment for its efficient incorporation into virions. J Virol.

[12] Gao Q, Palese P (2009). Rewiring the RNAs of influenza virus to prevent reassortment. Proc Natl Acad Sci U S A.

[13] Hutchinson EC, Curran MD, Read EK, Gog JR, Digard P (2008). Mutational analysis of cis-acting RNA signals in segment 7 of influenza a virus. J Virol.

[14] Marsh GA, Hatami R, Palese P (2007). Specific residues of the influenza a virus hemagglutinin viral RNA are important for efficient packaging into budding virions. J Virol.

[15] Marsh GA, Rabadan R, Levine AJ, Palese P (2008). Highly conserved regions of influenza a virus polymerase gene segments are critical for efficient viral RNA packaging. J Virol.

[16] Muramoto Y, Takada A, Fujii K, Noda T, Iwatsuki-Horimoto K, Watanabe S, Horimoto T, Kida H, Kawaoka Y (2006). Hierarchy among viral RNA (vRNA) segments in their role in vRNA incorporation into influenza a virions. J Virol.

[17] Chou YY, Vafabakhsh R, Doganay S, Gao Q, Ha T, Palese P (2012). One influenza virus particle packages eight unique viral RNAs as shown by FISH analysis. Proc Natl Acad Sci U S A.

[18] Inagaki A, Goto H, Kakugawa S, Ozawa M, Kawaoka Y (2012). Competitive incorporation of homologous gene segments of influenza a virus into virions. J Virol.

[19] Gerber M, Isel C, Moules V, Marquet R (2014). Selective packaging of the influenza a genome and consequences for genetic reassortment. Trends Microbiol.

[20] Markowska-Daniel I, Stankevicius A (2005). Seroprevalence of antibodies against swine influenza viruses in pigs of different age. Bull Vet Inst Pulawy.

[21] Hoffmann B, Harder T, Lange E, Kalthoff D, Reimann I, Grund C, Oehme R, Vahlenkamp TW, Beer M (2010). New real-time reverse transcriptase polymerase chain reactions facilitate detection and differentiation of novel A/H1N1 influenza virus in porcine and human samples. Berl Munch Tierarztl Wochenschr.

[22] EJA S, RAM F. Host adaptation and transmission of influenza a viruses in mammals. Emerg Microbes Infect. 2014; doi:10.1038/emi.2014.9. 10.1038/emi.2014.9 PMC3944123 26038511

[23] Su S, Bi Y, Wong G, Gray GC, Gao GF, Li S (2015). Epidemiology, evolution, and recent outbreaks of avian influenza virus in China. J Virol.

[24] Su S, Zhou P, Fu X, Wang L, Hong M, Lu G, Sun L, Qi W, Ning Z, Jia K, Yuan Z, Wang H, Ke C, Wu J, Zhang G, Gray GC, Li S (2014). Virological and epidemiological evidence of avian influenza virus infections among feral dogs in live poultry markets, china: a threat to human health?. Clin Infect Dis.

[25] Chou YY, Albrecht RA, Pica N, Lowen AC, Richt JA, García-Sastre A, Palese P, Hai R (2011). The M segment of the 2009 new pandemic H1N1 influenza virus is critical for its high transmission efficiency in the guinea pig model. J Virol.

[26] Ince WL, Gueye-Mbaye A, Bennink JR, Yewdell JW (2013). Reassortment complements spontaneous mutation in influenza a virus NP and M1 genes to accelerate adaptation to a new host. J Virol.

[27] Campbell PJ, Danzy S, Kyriakis CS, Deymier MJ, Lowen AC, Steel J (2014). The M segment of the 2009 pandemic influenza virus confers increased neuraminidase activity, filamentous morphology, and efficient contact transmissibility to A/Puerto Rico/8/1934-based reassortant viruses. J Virol.

[28] Jackson S, Van Hoeven N, Chen LM, Maines TR, Cox NJ, Katz JM, Donis RO (2009). Reassortment between avian H5N1 and human H3N2 influenza viruses in ferrets: a public health risk assessment. J Virol.

[29] Shortridge KF, Zhou NN, Guan Y, Gao P, Ito T, Kawaoka Y, Kodihalli S, Krauss S, Markwell D, Murti KG, Norwood M, Senne D, Sims L, Takada A, Webster RG (1998). Characterization of avian H5N1 influenza viruses from poultry in Hong Kong. Virology.

[30] Chen LM, Davis CT, Zhou H, Cox NJ, Donis RO. Genetic compatibility and virulence of reassortants derived from contemporary avian H5N1 and human H3N2 influenza a viruses. PLoS Pathog. 2008; doi:10.1371/journal.ppat.1000072. 10.1371/journal.ppat.1000072 PMC2374906 18497857

[31] Ma W, Lager KM, Lekcharoensuk P, Ulery ES, Janke BH, Solórzano A, Webby RJ, García-Sastre A, Richt JA (2010). Viral reassortment and transmission after co-infection of pigs with classical H1N1 and triple-reassortant H3N2 swine influenza viruses. J Gen Virol.

[32] Van Riel D, Munster VJ, De Wit E, Rimmelzwaan GF, Fouchier RA, Osterhaus AD, Kuiken T (2007). Human and avian influenza viruses target different cells in the lower respiratory tract of humans and other mammals. Am J Pathol.

