# Supplementary material for: Reassortment process after co-infection of pigs with avian H1N1 and swine H3N2 influenza viruses
Source: BMC Vet Res. 2017 Jul 8;13:215. doi: 10.1186/s12917-017-1137-x (PMC5501944; doi:10.1186/s12917-017-1137-x)
Supplement: Additional file 1: Table S1. — Contains the names and sequences of all primers used to amplify fragments of all genes to differentiate gene segments of the Gent/08 and the Italy/05. Table S2. Contains the information on which gene of which strain (Italy/05 or Gent/08) is subject to restriction enzyme cleavage. It also provides the information about the size of the RT-PCR products and the fragments after the restriction enzyme digestion. (DOCX 14 kb) [file 12917_2017_1137_MOESM1_ESM.docx]

Table S1. Primers for RT-PCRs for molecular differentiation of the Gent/08 and the Italy/05

| RT-PCR | Name of primer | Primer sequence (5’-3’) | Gene |
| --- | --- | --- | --- |
| 1 | PB2-F | TCGAGGTATTGCATTTGACTC | PB2 |
|  | PB2-1366R | ATCAATAGGTTCAATTCCCC |  |
| 2 | PB1-F | GTTCATCAAGGACTACAGATA | PB1 |
|  | PB1-R | TCTGAGCTCTTCAATGGTGG |  |
| 3 | PA-F | GTCTCCCGCCTAACTTCTCC | PA |
|  | PA-R | TCCAGCTTGCCAGTGATCTA |  |
| 4 | HA-Gent-F | TGTTACCCTTATGATGTGCCC | HA |
|  | HA-Gent-R | GGTTTCCATTGCTGTTGATT |  |
| 5 | HA-Italy-F | GCTGCTCAATGCGAGTTCAT |  |
|  | HA-Italy-R | GCACTTCTTTTCCCTTGTTGTT |  |
| 6 | NP-F1 | CCAGAACAGCATAACAATAGAGAGA | NP |
|  | NP-F2 | CCAAAATAGTATAACGATAGAGAGA |  |
|  | NP-R | TACACACAAGCAGGCAAGCA |  |
| 7 | NA-Gent-F | CCACAATGCAAAATCACAGG | NA |
|  | NA-Gent-R | CCAAATGAAATGGAACACCC |  |
| 8 | NA-Italy-F | GGTTCCAAAGGGGATGTATTT |  |
|  | NA-Italy-R | CTTGATAGTGTCCGTTATTATGCC |  |
| 9 | M-1F | AGCAAAAGCAGGTAG | M |
|  | M-1027R | AGTAGAAACAAGGTAGTTTTT |  |
| 10 | NS-F | GTGATGCCCCATTTCTTGAT | NS |
|  | NS-R | ATGTCCTTATCTCTTGCTCCAC |  |

Table S2. Differentiation of the Italy/05 and the Gent/08 by the RT-PCR and the RFLP analyses

| **Virus** | **Gene** | | **Gene/enzyme** | | | | | |
| --- | --- | --- | --- | --- | --- | --- | --- | --- |
|  | HA | NA | PB2/*Eco*RI | PB1/*Bam*HI | PA/*Bam*HI | NP/*Bam*HI | M/*Nhe*I | NS/*Bam*HI |
| **Italy/05** | 314 | 463 | 281  399 | 266  348 | 570 | 670 | 1027 | 736 |
| **Gent/08** | 327 | 241 | 680 | 614 | 227  343 | 307  363 | 457  570 | 282  454 |

Fragment sizes are given in bp.
